# Fertility Impairment after Trekking at High Altitude: A Proof of Mechanisms on Redox and Metabolic Seminal Changes

**DOI:** 10.3390/ijms23169066

**Published:** 2022-08-13

**Authors:** Vittore Verratti, Simona Mrakic-Sposta, Jonathan Fusi, Iva Sabovic, Ferdinando Franzoni, Tiziana Pietrangelo, Danilo Bondi, Stefano Dall’Acqua, Simona Daniele, Giorgia Scarfò, Camillo Di Giulio, Andrea Garolla

**Affiliations:** 1 Department of Psychological, Health and Territorial Sciences, University “G. D’Annunzio” of Chieti-Pescara, 66100 Chieti, Italy; 2 Institute of Clinical Physiology, National Research Council (IFC-CNR), 20162 Milan, Italy; 3 Department of Clinical and Experimental Medicine, University of Pisa, 56126 Pisa, Italy; 4 Department of Medicine, University of Padova, 35122 Padova, Italy; 5 Department of Neuroscience, Imaging and Clinical Sciences, University “G. D’Annunzio” of Chieti-Pescara, 66100 Chieti, Italy; 6 Department of Pharmaceutical and Pharmacological Science, University of Padova, Via Marzolo 5, 35128 Padova, Italy; 7 Department of Pharmacy, University of Pisa, 56126 Pisa, Italy

**Keywords:** altitude hypoxia, male infertility, redox biology, oxidative stress, sperm parameters, sperm physiology

## Abstract

Many authors described negative but reversible effects of high-altitude hypoxic exposure on animal and human fertility in terms of sperm concentration, function, and biochemical alterations. The aim of this study was to evaluate the acute and chronic effects of high-altitude exposure on classical sperm parameters, redox status, and membrane composition in a group of travellers. Five healthy Italian males, all lowlanders not accustomed to the altitude, were evaluated after 19 days-trekking through low, moderate, and high altitudes in the Himalayas. Sperm samples were collected before (*Pre*), 10 days after (*Post*), and 70 days after the end of the expedition (*Follow-up*). Sperm concentration, cholesterol and oxysterol membrane content, and redox status were measured. Hypoxic trek led to a significant reduction in sperm concentration (*p* < 0.001, η^2^p = 0.91), with a reduction from *Pre* to *Post* (71.33 ± 38.81 to 60.65 ± 34.63 × 10^6^/mL) and a further reduction at *Follow-up* (to 37.13 ± 39.17 × 10^6^/mL). The seminal volume was significantly affected by the hypoxic trek (*p* = 0.001, η^2^p = 0.75) with a significant reduction from *Pre* to *Post* (2.86 ± 0.75 to 1.68 ± 0.49 mL) and with partial recovery at *Follow-up* (to 2.46 ± 0.45 mL). Moreover, subjects had an increase in ROS production (+86%), and a decrease in antioxidant capacity (−37%) in the *Post* period with partial recovery at *Follow-up*. These results integrated the hormonal response on thyroid function, hypothalamus–pituitary–gonadal axis, and the prolactin/cortisol pathways previously reported. An uncontrolled ROS production, rather than a compromised antioxidant activity, was likely the cause of impaired sperm quality. The reduction in fertility status observed in this study may lie in an evolutionary Darwinian explanation, i.e., limiting reproduction due to the “adaptive disadvantage” offered by the combined stressors of high-altitude hypoxia and daily physical exercise.

## 1. Introduction

### 1.1. High-Altitude Physiology

Compared with other areas of research such as cardiovascular [1] and respiratory physiology [2], as well as endocrine [3], metabolic [4,5], muscular, and exercise physiology [6,7], few studies are present in the literature regarding physiological adaptations at high-altitude hypoxia related to male fertility status. There are several inhabited regions of the planet at high altitudes, as well as those places of tourism where trekking, exploration, and alpinism are present. If one considers the Himalayas and Andes as the only high-altitude regions of native populations [8,9], human chronic exposure to high altitudes is an exceptional situation, which reaffirms the reputation of high altitudes as “extraordinary environments”. In this regard, for the physical–environmental characteristics, high altitude is the natural laboratory to study human physiological response to hypoxic conditions [10,11].

From a biological and evolutionary point of view, the adaptability of physiological processes that guarantee male reproductive homeostasis, among the various difficulties presented by the external environment, must inevitably overcome the obstacle present in the nature of high-altitude hypoxia.

### 1.2. Male Reproductive Physiology at High Altitude

Historically, pioneering scientific frames emerge showing how the first observations of high-altitude effects on the state of fertility derive from observations on the reproductive capacity of animals brought from maritime regions at high altitudes [12], but also from human migration, mostly of a colonial nature, to the high lands of South America. Of scientific and historical interest are the reports of an Augustinian friar, Antonio de la Calancha, describing in 1638 a long reproductive stop among the Spanish (conquistadores) established in Potosi at 4267 m (Bolivia), highlighting a process of reproductive adaptation over a period of 50 years compared with an indigenous reproductive average of 100%. He also described an important reduction in reproductive capacity in imported animals (horses, chickens, and pigs) in Jauja (3962 m), Peru’s first capital city (de la Calancha, 1972 [13]). Of interest are the first scientific observations of Carlos Monge, father of Andean Mountain medicine, reporting that only 60% of rams, brought from sea level at high altitude, can procreate during the first year, with a reproductive average of 70% after two or three years, reaching to a “restitutio ad integrum” of reproductive function in the acclimatised ram. Similar disorders, as Monge affirmed, also occur in rabbits, cats, horses, and cattle but also in humans with various pathological conditions of impaired spermatogenesis, similar to those of cryptorchidism [14].

With the progress of technological–investigative resources, the scientific knowledge in this research field has made considerable acquisitions while not betraying the intuitions in the epistemological discussion of pioneers on the relationship between male fertility and high-altitude hypoxia. In a cross-sectional study on sperm quality, Zou’s research group [15] showed that soldiers assigned to high-altitude areas (Lhasa, at 3700 m of altitude) presented semen volume, total sperm count, and rapid progressive motility significantly lower than those of other centres and those of the WHO’s recommendations [16]. Some authors affirmed that high-altitude hypoxia causes sperm DNA damage [17], associated with reduced concentration and sperm apoptosis [17]. A study by He et al. showed that high-altitude exposure generates reversible alterations of semen parameters in humans at 6 months (total count, density, motility, and sperm survival rate decreased with liquefaction time prolonged), with partial adaptation at 12 months (total count and sperm density normalised) and complete recovery at 6 months from their return to sea level [18]. Similar results were obtained by our group describing a complete normalisation of the seminal state in humans, characterised by oligospermia, a reduction in motility, the total number of motile sperm, and increased dimorphisms, induced by 26 days spent at high altitudes (from 2000 to 5600 m; Karakorum expedition) [19]. In line with the latter study, Zheng et al. affirmed that after 3 months of permanence at 3600 m (Lhasa City, Tibet), a reduction in sperm concentration with changes in the distribution of sperm dysmorphisms, characterised by an increase in the rate of head malformation, occurs in humans [20]. Comparing the seminal characteristics of male Tibetan high-altitude natives with those of male “Tibetan Han” high-altitude immigrants, a capacity for reproductive adaptation to the high-altitude hypoxia of the first ones emerged; instead, adaptive alterations in concentration and sperm motility, characterising states of severe azoospermia and oligozoospermia were found in the Tibetan Han exposed to high altitudes [21].

Several studies investigated the effects of high-altitude hypoxia on male reproductive physiology in animal models. According to Cofrè et al., the reproductive efficiency of rams born at low altitudes but raised and bred at high altitudes is lower than those of rams born and bred at low altitudes. This phenomenon may be due to the hypoxic and oxidative stresses that generate a decrease in concentration, progressive motility, and viability of spermatozoa, as well as a decrease in the antioxidant state of seminal fluid and increased oxidative blood stress [22]. Studies in rats exposed to chronic and intermittent hypobaric hypoxia revealed changes in testicular morphology combined with a loss of seminiferous cells, in all stages of the spermatogenic cycle [23]. Extending these considerations on morphological changes in response to hypoxic stress and confirming the data obtained from animals [23], our group described, beyond the characteristic alterations of sperm parameters [3], a reduction in total testicular volume in humans [24].

### 1.3. Redox Role

As is well known, at high altitudes, the increase in reactive oxygen species (ROS), such as the one produced in response to a hypoxic state [10], induces generalised damage able to affect the fundamental cellular components such as carbohydrates, proteins, lipids and DNA [25], and seems to result in increased apoptosis at the germ cell level [26]. This phenomenon results, overall, in a state of hypospermatogenesis, critical for the viability and quality of reproductive cells and for the global state of male fertility [26]. However, as it is not very clear if high altitude is an oxidative stress generator in spermatozoa, the use of the animal model is acceptable, if not obligatory, from the point of view of a desirable advancement of knowledge concerning the damage generated by oxidative metabolism in the male gametes, to understand the mechanistic basis of sperm function [26]. This aspect is very relevant in human and animal reproduction because the imbalance between the ROS and antioxidant capacity (TAC) in semen indicates oxidative stress, and it is strictly related to male infertility [27].

### 1.4. The Role of Cholesterol and Its Metabolites

Sperm membrane fluidity, capacitation, and acrosomal reaction are strongly linked with cholesterol, and sperm capacitation is associated with oxysterols as the oxidised products of cholesterol [28]. Moderate oxysterol concentrations are involved in the regulation of sperm capacitation induced by ROS. However, their concentration is enriched in the pathological conditions of uncontrolled lipid peroxidation, such as from impairment of the cell redox balance, which generates toxicant oxysterol species such as 7-β-hydroxycholesterol (7b-OHC) and 7-ketocholesterol (7-KC), thus impairing the capacitation process [29].

### 1.5. Purpose

The aim of this experimental project was to investigate and mark a new point on male fertility by means of metabolic and oxidative stress analysis on the spermatic samples obtained by Italian explorers exposed to trekking under hypoxia.

## 2. Results

### 2.1. Sperm Parameters

**Figure 1:**
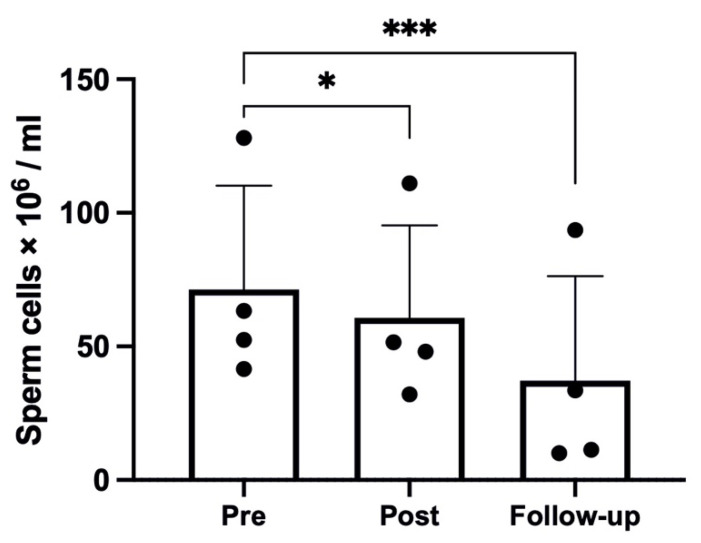
Sperm count results; one participant was removed from the analysis due to too low values; changes over time were significant at * *p* < 0.05; *** *p* < 0.001.

The hypoxic trek led to a significant reduction in sperm concentration (*p* < 0.001, η^2^_p_ = 0.91, ω^2^_p_ = 0.88), with a reduction from *Pre* to *Post* (71.33 ± 38.81 to 60.65 ± 34.63 × 10^6^/mL, *p* = 0.025), as shown in Figure 1, and a further reduction at *Follow-up* (to 37.13 ± 39.17 × 10^6^/mL, *p* < 0.001 compared with *Pre*). The seminal volume was significantly affected by the hypoxic trek (*p* = 0.001, η^2^_p_ = 0.75, ω^2^_p_ = 0.70), with a significant reduction from *Pre* to *Post* (2.86 ± 0.75 to 1.68 ± 0.49 mL, *p* = 0.001) and partial recovery at *Follow-up* (to 2.46 ± 0.45 mL, *p* = 0.013).

### 2.2. Cholesterol and Oxysterol Membrane Content

**Figure 2:**
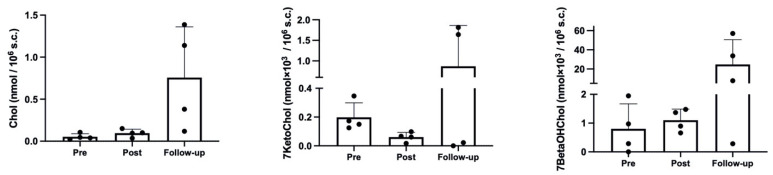
Cholesterol and oxysterol results.

As shown in Figure 2, the hypoxic trek led to a significant increase in cholesterol (*p* = 0.048, η^2^_p_ = 0.54, ω^2^_p_ = 0.41), with a clear, although non-significant in post hoc comparisons, increase at *Follow-up*, compared with both *Pre* (*p* = 0.083) and Post (*p* = 0.105). The effect on 7-ketocholesterol was not significant (*p* = 0.168, η^2^_p_ = 0.33, ω^2^_p_ = 0.17), although it showed a decrease at *Post* and an increase over baseline values at *Follow-up*. A similar result to cholesterol was obtained for 7-beta hydroxycholesterol (*p* = 0.108, η^2^_p_ = 0.43, ω^2^_p_ = 0.28), with almost similar values indicating a slight increase at *Post*, compared with *Pre*, and a further increase at *Follow-up*.

### 2.3. Redox Analysis

**Figure 3:**
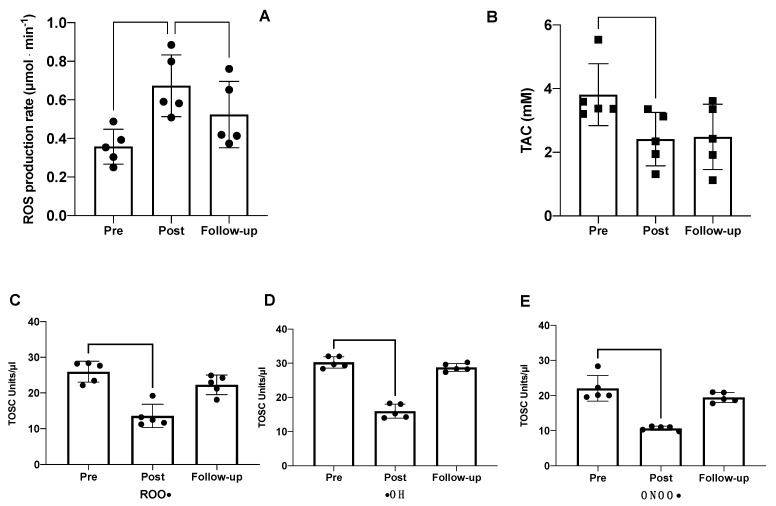
EPR results of (**A**) ROS production rate and (**B**) TAC via EPR detection. ORAC results of (**C**) anti-peroxyl (ROO•), (**D**) anti-hydroxyl (•HO), and (**E**) anti-peroxynitrite (ONOO•) antioxidant activity of human seminal fluid. Significantly different data are displayed between brackets. Changes over time were significant at * *p* < 0.05; ** *p* < 0.01.

The ROS production rate and total antioxidant capacity measured in seminal fluids are shown in Figure 3. The hypoxic trek led to significant changes in the oxidative stress balance. Specifically, compared with the baseline measurements at sea level (pre-expedition), the seminal fluid production rate significantly increased post-expedition (0.36 ± 0.09 vs. 0.67 ± 0.16 μmol × min^−1^), with a trend to return toward baseline levels thereafter (0.67 ± 0.16 vs. 0.52 ± 0.17 μmol × min^−1^) (Figure 3A). Conversely, the TAC was significantly decreased at *Post* and *Follow-up* (3.80 ± 0.97 vs. 2.41 ± 0.84 mM) compared with *Pre* (Figure 3B).

**Figure 4:**
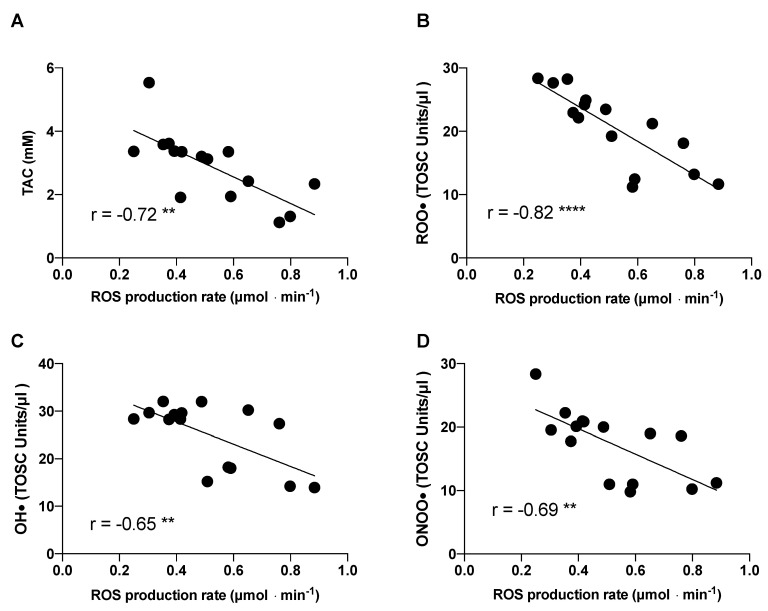
Multipanel plots of ROS production rate levels relative to (**A**) TAC, (**B**) ROO•, (**C**) OH•, and (**D**) ONOO•. The linear regression fit (solid line) is also shown and so is the correlation coefficient (r) reported in each panel. A significant linear relationship was estimated as ** *p* < 0.01; **** *p* < 0.001.

The evaluation of the specific antioxidant activity of seminal fluids was performed using the TOSC assay. As depicted in Figure 3, the semen collected at *Post* showed a significant decrease in the scavenging activity against all three radical species compared with *Pre*: peroxyl, hydroxyl, and peroxynitrite derivatives. These results suggest that following exposure to high altitudes, the semen is more easily attacked by radical species, leading to increased damage in terms of motility and fertility. An increase in semen antioxidant activity against the three radical species was observed at *Follow-up*, indicating the partial recovery of the antioxidant capability after 70 days from the end of the expedition. Linear regression analysis, shown in Figure 4, revealed negative associations between ROS production rate and TAC, peroxyl, hydroxyl, and peroxynitrite derivatives.

### 2.4. Other Redox and Hormonal Results

Regarding blood redox analyses, as reported, post-trek increases in ROS production rate, NOx, total Hcy, and GSH were detected; conversely, TAC and total Cys and CysGly decreased at *Post* [30]. The key result concerning the endocrine system was the impairment of the hypothalamic–hypophyseal axis. On the one hand, no difference was found in FSH and LH concentrations. The total testosterone decreased after the trek (from 4.86 ± 1.68 to 4.35 ± 0.95 ng/mL). Similarly, 17-β-oestradiol and prolactin were reduced after the trek. Regarding the thyroid axis, TSH did not change, while fT3 diminished after the trek [31].

## 3. Discussion

Oxidative stress (OxS) is defined as the excessive production of ROS related to antioxidant defence and can be triggered by endogenous and exogenous factors, among which are expeditions to high altitudes. In fact, exposure to high altitude has been associated with an increase in ROS and related oxidative damage [10,11,30,32] induced by environmental factors such as hypoxia, cold, UV ray, exposure, and/or physical exercise (daily frequency, low-middle intensity, high volume) [30]. Moreover, Agarwal et al. reported that several extrinsic or environmental factors, such as ionising radiations, alcohol, obesity, deficiencies in antioxidants, varicocele, bacterial/viral infections, toxins, and chemotherapy can induce testicular ROS, causing abnormal spermatogenesis [33]. The burden of oxidative stress increases during the time spent at altitude and may even persist for some time upon return to sea levels. In fact, the adaptive process requires a relatively long period of time, as previously reported [10,32,34].

Although low/moderate concentrations of ROS are essential for several functions such as the host defence system and the regulation of various intracellular signalling cascades [35], spermatozoa are more vulnerable than other cells to the overproduction of ROS due to the elevated levels of polyunsaturated fatty acids (i.e., docosahexaenoic acid) in their plasma membrane [36]. As also reported in the literature, changes in the redox status of spermatozoa lead to the generation of ROS [37]. Therefore, an optimal level of ROS is essential for maintaining spermatogenesis acrosome reaction, sperm motility/functions, and hence fertility [38].

Furthermore, the OxS in sperm can arise from intrinsic sources such as damaged sperm and/or ageing [39] or extrinsic factors such as lifestyle and environmental exposure [40]. These observations are in accordance with the measurements that we collected from our subjects. Post-expedition, we found an increase of 86% of seminal ROS with a consequent decrease of 37% in TAC. As reported by Sikka [41], uncontrolled ROS production has a detrimental effect on the function and quality of sperm; as a matter of fact, high levels of ROS, rather than a compromised antioxidant activity, are major causes of oxidative-stress-induced male infertility [33,42]. Furthermore, antioxidant molecules may alter spermatozoa maturation, interfering with physiological sperm function, and the lack of cytoplasmic antioxidant enzymes makes them highly susceptible to oxidation [43], with consequent detrimental effects on sperm quality and function. Many studies reported that the increase in ROS can lead the disequilibrium in the antioxidant capacity and then cause damage to the sperm membrane, loss of membrane integrity and increased permeability, lipid peroxidation, germ cell DNA damage, and apoptosis, leading to sperm with abnormal morphology, DNA damage [44], increased apoptosis [45], declined motility, and low sperm concentrations [46].

ROS are a broad range of radicals, such as hydroxyl ion [OH−], superoxide ion [O_2_−], nitric oxide [NO], peroxyl [RO_2_], lipid peroxyl [LOO], and non-radical molecules, namely singlet oxygen [−1O_2_], hydrogen peroxide [H_2_O_2_], lipid peroxide [LOOH], and ozone [O_3_]. Our data showed that total oxidative scavenging capacity vs. ROO^•^, •OH, and ONOO• decreased, respectively, −46, −52, and −48% after the expedition, but the values were restored at 70 days of follow-up.

The overproduction of ROS after the expedition was parallel with the decrease in sperm concentration −15% at *Post*, which was further decreased down to −48% after 70 gg. These data are very important considering that normally, this parameter ranges from 15 to 259 million per mL and that a sperm count below 39 million sperm per ejaculate is considered low [47]. At *Pre*, our subjects showed a value between 65 and 75 million per mL, falling within the normal values, but especially in the *Follow-up* period, the concentrations dramatically decreased. This is probably related to the hypoxic status, physical exercise, and several environmental adverse conditions during the 300 km expedition in the Kanchenjunga region for 19 days.

**Figure 5:**
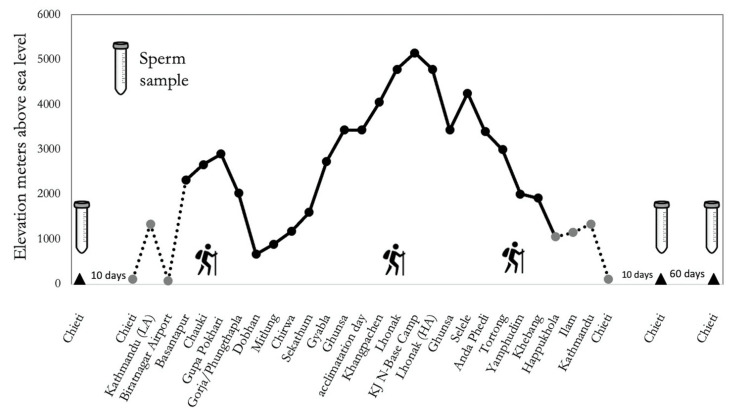
Study design of “Kanchenjunga Exploration and Physiology” project.

Male fertility is essentially based on the constant production of spermatozoa, generated from spermatogonial stem cells (SSCs) through their self-renewal and by the processes of mitosis, meiosis, and spermiogenesis, leading to mature sperm [48] with a production cycle of approximately 70 days [49]. This was the reason for determining the follow-up period as after 70 days, which can explain the further fall in sperm count. Such reduced sperm count at *Follow-up*, along with the increase in cholesterol and oxysterols, can be interpreted mainly as the effect of the redox system disruption that occurred during the expedition, due to hypoxic stress on the testis parenchyma and more precisely at the germinative epithelium of the seminiferous tubules. This stress was present during the first and proliferative phase of spermatogenesis which lasts 16 days. Precisely, during these 16 days of the “mitotic phase”, spermatogonial stem cells generate cells that undergo amplifying divisions and change into differentiating spermatogonia [50]. In other terms, the entire period of hypoxic trekking (see Figure 5) coincided with the entire first proliferative phase of spermatogenesis. Therefore, it is reasonable to argue that the negative effects on the seminal profile showed the greater involvement of the mitotic processes of spermatogenesis rather than those related to the meiotic and/or spermiogenesis phases.

In relation to the seminal volume, the reduction at *Post* was almost fully recovered at *Follow-up*. As is known, volume is very largely determined by seminal plasma, a nutritive-protective fluid produced in the rete testis, epididymis, and the accessory sex glands, as a vehicle for transporting ejaculated spermatozoa, which constitute only 10% of the total volume [51]. Therefore, it is reasonable to assume that the reduction we observed was determined, rather than a disruption in the production processes, by a loss of bodily fluid and sodium triggered by augmented diuresis and natriuresis commonly induced at high altitudes [52]. Indeed, previous results have shown a loss of bodily fluids in the participants of the “Kanchenjunga Exploration and Physiology” project through a series of bioimpedance analyses during the expedition [53]. The recovery of volume at *Follow-up* supports this physiological interpretation. Along with the herein described insights, the previously reported hormonal results strengthen the concept of a worsening of the fertility system. Considering the framework of redox system disruption and further male fecundity impairment triggered by hypoxia, we support the use of redox testing in male fertility evaluations, as recently highlighted by a global survey [54]. Future studies with larger samples may unveil novel insights by linking the investigation of this study with epigenetic changes.

It is worth recalling that life on Earth has spread through adaptations and natural selection, within certain limits, as reported by Darwin’s empirical observations in his renowned book *On the Origin of Species*:

*“What checks the natural tendency of each species to increase in number is most obscure [...] Each species, even where it most abounds, is constantly suffering enormous destruction at some period of its life, from enemies or from competitors for the same place and food [...] When we travel southward and see a species decreasing in numbers, we may feel sure that the cause lies quite as much in other species being favoured, as in this one being hurt. So it is when we travel northward, but in a somewhat lesser degree, for the number of species of all kinds, and therefore of competitors, decreases northwards; hence in going northward, or in ascending a mountain, we far oftener meet with stunted forms, due to the directly injurious action of climate, than we do in proceeding southwards or in descending a mountain. When we reach the Arctic regions, or snow-capped summits, or absolute deserts, the struggle for life is almost exclusively with the elements”*

[55]

Therefore, although the current study did not investigate an evolutionary model, the results can be seen under an evolutionary, Darwinian explanation, i.e., limiting reproduction due to the “adaptive disadvantage” in terms of long-term survival triggered by combining exposure to high-altitude hypoxia with daily low-to-medium intensity exercise. In addition to hypoxia, the selection triggered by the harsh environment of high altitude had been influenced by other environmental factors such as cold and limited food sources, and strongly by behavioural determinants [56].

## 4. Materials and Methods

### 4.1. Participants and Study Protocol

A group of 5 healthy Italian males, all lowlanders not accustomed to altitude (see Table 1) participated in this trial/trekking in the context of the research project “Kanchenjunga Exploration and Physiology”, which represents a subset of a wider project approved by the Ethical Review Board of the Nepal Health Research Council (NHRC). All study procedures were performed according to the ethical standards of the 1964 Helsinki declaration and later amendments. All participants signed their written informed consent. The study was conducted in accordance with the STAR Data Reporting Guidelines for Clinical High-Altitude Research [57]. Participants completed a circuit of 300 Km distance in 19 days (see Figure 5), with over 16,000 m of difference in altitude, covering a daily average walk of 6 h. The route involved demanding ascent and descent in the Kanchenjunga region, Himalayas, Nepal.

The expedition was supervised by a trained medical doctor, who was monitoring symptoms, SpO_2_, blood pressure, and anthropometric data throughout the whole period. None of the participants suffered from acute mountain sickness.

### 4.2. Semen Collection and Analysis

Semen samples were collected at three different time points: before the expedition, after 10 days, and after 70 days, as the duration of spermatogenesis in humans is reported about 70 days [49]. Ejaculates were collected in a sterile recipient by masturbation after 3–4 days of abstinence. Semen samples were allowed to liquefy for 30 min and were quantitatively examined for sperm count; diagnostic tests of routine semen analysis in clinical andrology were conducted according to the WHO criteria [16]; the results were used as inclusion criteria for further analyses. After semen evaluation, samples were washed twice in phosphate-buffered saline (PBS), and the cell pellet was stored at −80 °C until use.

### 4.3. Extraction and Cholesterol/Oxysterols Measurements Using LC-MS

The total lipid fraction was extracted from the pellet of sperm cells with a chloroform–methanol mixture [58]. Briefly, the sperm cell pellet was incubated with 1 mL of the chloroform–methanol mixture under gentle mixing at 37 °C. The subsequent dilution with chloroform and water separated the extract into two layers with the chloroform layer at the bottom containing the lipids and the hydroalcoholic layer at the top containing all the non-lipidic species. The chloroform layer was separated, transferred to Eppendorf, and lyophilised in a Vacufuge^®^-Concentrator plus (Hamburg, Germany). The residue was resolubilised in 200 µL of chloroform, underwent isolation of the sterol fraction via solid-phase extraction (SPE) on a 1 mL silica column, and preconditioned with a methanol–chloroform (1:1 *v*/*v*) solution, through elution with acetone. The lyophilised sterol fraction was dissolved in methanol, and 20 µL was injected into a high-performance liquid chromatography–mass spectrometry system composed of an Agilent 1260 binary pump, a Varian 430 autosampler, a column oven, and a Varian 320MS triple quadrupole mass spectrometer. A Zorbax eclipse plus C18 column (2.1 mm × 50 mm, 3.5 µm particle size; Agilent, Palo Alto, CA, USA) was used at the temperature of 40 °C for the chromatographic step. Isocratic elution was performed using a mixture of mobile phase A: H_2_0 MilliQ + 0.1% formic acid (A) and methanol + 0.3% formic acid (B) in equal parts (*v*/*v*). The positive ionisation of the eluted sample was performed via electrospray ionisation with a capillary potential of 4.5 kV. The source chamber’s temperature was 50 °C, that of the drying gas was 265 °C, and the temperature of the vaporiser was 250 °C. The quantification of cholesterol, 7-ketocholesterol, and 7-β-cholesterol was performed using the corresponding calibration curves prepared through serial dilution of each analyte. For cholesterol, the selected transition was *m/z* 369 > 287, while for 7-ketocholesterol, it was 401.43 > 95.05 and for 7-β-cholesterol, 367 > 147. Calibration curves were obtained using the reference compounds in the concentration levels of 0.010, 0.025, 0.050, 0.075, 0.1, 0.25, 0.5, 0.75 and 1 µg mL^−1^. The LOD and LOQ values were 32 ng/mL and 96 ng/mL for cholesterol and 25 ng/mL and 75 ng/mL for 7-ketocholesterol and 7-β-cholesterol, respectively. The data were acquired using the Varian Workstation (6.12.3) software and subsequently expressed as nanomoles per 10^6^ sperm cells and were the averages of three independent replicates ± standard deviation.

### 4.4. ROS and TAC Determination via Electron Paramagnetic Resonance

The ROS production rate and antioxidant capacity were determined using the consolidated method [10,32,59,60,61,62]. An EPR instrument (E-scan-Bruker BioSpin GmbH, Billerica, MA, USA) X-band (9.3 GHz) was utilised. A CMH (1-hydroxy-3-methoxycarbonyl-2,2,5,5-tetramethylpyrrolidine) probe was used for ROS detection in seminal fluid, and a stable radical CP• (3-carboxy2,2,5,5-tetramethyl-1-pyrrolidinyloxy) was used as an external reference in order to convert ROS in absolute quantitative values (µmol × min^−1^), while DPPH• (2,2-diphenyl-1-picrylhydrazyl) was used to measure antioxidant capacity.

Samples were stabilised at 37 °C using a Temperature and Gas Controller ‘‘Bio III’’ unit (Noxigen Science Transfer and Diagnostics GmbH, Elzach, Germany), interfaced with EPR. The Bruker software was adopted for spectra acquisition and handling (Win EPR System, V. 2.11; Billerica, MA, USA).

### 4.5. Total Oxyradical Scavenging Capacity Assay (TOSCA)

The antioxidant scavenging capacity of seminal plasma and sperm cell fractions toward peroxyl radicals (ROO), hydroxyl radicals (OH), and peroxynitrite (HOONO) was measured with a total oxyradical scavenging capacity assay (TOSCA) [63,64,65]. Peroxyl, hydroxyl, and peroxynitrite radicals were produced through the thermal homolysis of 2-2′-azo-bis-(2 methylpropionamidine)-dihydrochloride (ABAP), an iron–ascorbate Fenton-like reaction and from 3-morpholinosydnonimine hydrochloride (SIN-1), respectively, as follows: (a) 0.2 mM KMBA, 20 mM ABAP in a 100 mM potassium phosphate buffer, pH 7.4 for peroxyl radicals; (b) 1.8 µM Fe3 +, 3.6 µM EDTA, 0.2 mM KMBA, 180 µM ascorbic acid in a 100 mM potassium phosphate buffer, pH 7.4 for hydroxyl radicals; (c) 0.2 mM KMBA and 80 µM SIN-1 in a 100 mM potassium phosphate buffer, pH 7.4 with 0.1 mM diethylenetriaminepentaacetic acid (DTPA) for peroxynitrite.

In these assay conditions, the different oxyradicals induced a similar pro-oxidant pressure in the control reaction in terms of KMBA oxidation; the relative efficiency of a sample as a scavenger of different ROS could thus be compared by its ability to inhibit a quantitatively comparable ethylene formation but induced by different specific forms of oxyradicals. Reactions were conducted at 35 °C, and aliquots of 200 µL were taken every 10 min intervals for a total duration of 90 min. Ethylene production was measured with a Hewlett Packard (HP 4890 series) (Wilmington, DE, USA) gas chromatograph equipped with a Supelco SPB-1 capillary column (30 m × 0.32 mm × 0.25 µm) (Supelco, Bellefonte, PA, USA) and a flame ionisation detector.

For the various oxidant-generating systems, TOSC values were quantified from the following equation: TOSC = 100 − (∫SA/∫CA × 100), where ∫SA and ∫CA are the integrated areas calculated under the least squares kinetic curve produced during the reaction course for, respectively, sample (∫SA) and control (∫CA) reactions (23). A TOSC value of 0 (∫SA/∫CA = 1) indicates a sample with no scavenging capacity (i.e., no inhibition of ethylene formation), while a maximum theoretical TOSC value of 100 corresponds to a total inhibition of ethylene formation throughout the assay (∫SA = 0). From the experimental TOSC values, for all the samples, a specific TOSC value relative to 1 mL of seminal liquid was calculated.

### 4.6. Other Markers from the Same Project

The blood redox analyses during this expedition have already been reported elsewhere [30]. The main results are reported since they are inherently linked to the redox network. Briefly, an X band electron paramagnetic resonance spectroscope (9.3 GHz) was used to assess the total ROS production and total antioxidant capacity of blood samples drawn from the antecubital vein two weeks before the start of the trip and the day after the end of the Himalayan trek.

The endocrine adaptations have also been reported elsewhere [31]; the main results are reported since they are inherently linked to the sexual axis. Briefly, the blood content of hormones was determined using an immuno-chemiluminescence assay.

### 4.7. Statistical Analysis

The statistical analysis was carried out using the R-based open-source software Jamovi Version 1.2.5.0 (retrieved from https://www.jamovi.org). The Shapiro–Wilk test was used for both the normality of distributions and the normality of residuals, Levene’s test was used for the equality of variances, and Q–Q plot observations were used for assumption checks. For spermatic count, we used the general linear mixed model (GLMM, with REML estimation and LRT as random effects and individuals as the random variables), to test the *Pre* vs. *Post* vs. *Follow-up* comparison. Post hoc tests were conducted with Bonferroni correction for multiple comparisons. The Satterthwaite method was used for degrees of freedom, and partial eta squared (η^2^_p_) and partial omega squared (ω^2^_p_) were calculated as measures of effect size. For redox analysis, we used repeated-measure ANOVA, after the assumption check. The relationship between selected variables was assessed using Pearson’s correlation coefficients. Data are expressed as mean ± SD. A *p* value < 0.05 was considered statistically significant. Graphs were created with GraphPad Prism Version 9 (GraphPad Software, La Jolla, CA, USA). Considering the type of study and its fieldwork nature, we performed a sensitivity analysis to calculate the minimum required effect size; given α = 0.05, 1 − β = 0.80, sample size = 5, number of measurements = 3, and an arbitrary correlation among repeated measures of 0.5, G*Power Version 3.1 ((https://www.psychologie.hhu.de/arbeitsgruppen/allgemeine-psychologie-und-arbeitspsychologie/gpower) computed a value of η^2^_p_ = 0.32.

## 5. Conclusions

**Figure 6:**
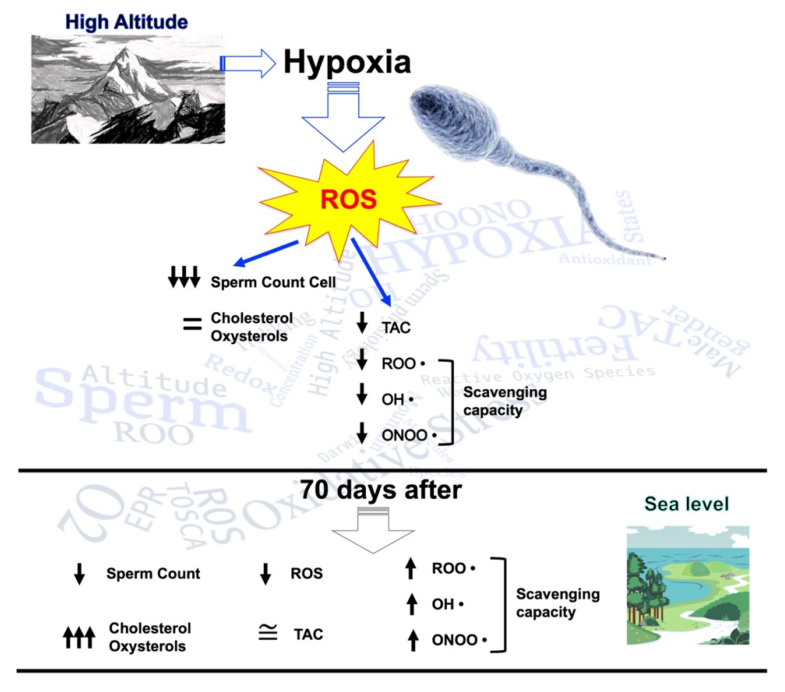
Scheme of the influence of high altitude on sperm count cells, cholesterol, oxysterols, reactive oxygen species (ROS) production, and antioxidant capacity. Evaluation was performed with seminal fluid collection.

Our results highlighted that hypoxic trekking triggers a disruption in the seminal redox network, along with the impairment of sperm quality, as shown early after the exposure by a reduction in sperm concentration and volume, and 70 days after by an increase in cholesterol and oxysterol levels (Figure 6).

In clinical andrology, sperm quality is used as a surrogate marker of male fecundity [66]. Although the WHO guidelines state 15 million spermatozoa per millilitre as the lowest limit for normal fertility [16], there is evidence of a progressively diminished male fecundity under 40 million spermatozoa per millilitre, and a window ranging from 15 to 40 million spermatozoa per millilitre of subfertility [67]. The reduction in sperm concentration we observed in our study, starting from 71.33 down to 60.65 at *Post* and 37.13 × 10^6^/mL at *Follow-up*, suggests a state of subfertility triggered by the hypoxic trekking model.

The low sample size represents the main limitation of this work, which should be attributed to its preliminary nature of a case series. In the same vein, the heterogeneity in age could have biased the results, as oxidative damage in sperm increases with age. Moreover, since the regimen of participants was similar during the expedition but likely not before and after the trek, additional information about the regimen, along with other respective environmental stressors of participants, would improve the insights in further larger sample studies. These weaknesses strongly limited the inferring of results and necessitate the use of caution in interpreting them. Prospectively, it would be interesting to compare the seminal characteristics of explorers already accustomed to altitude to assess an eventual reproductive adaptation process. In addition, analysing the sperm DNA damage and/or apoptosis would improve the comprehensive evaluation of sperm biology.

All in all, our pilot results revealed a likely burden in male physiological systems on maintaining an optimal fertile function in response to the combined stressors of altitude hypoxia and physical exercise. The impairment of fertility, along with the disruption of redox systems, may suggest a kind of brake on Homo sapiens’ reproductive capacity when exposed to high altitudes.

## Figures and Tables

**Table 1:** Descriptive of participants.

	Age (Years)	BMI Pre (kg/m^2^)	BMI Post (kg/m^2^)
KJ2	63	28.91	27.34
KJ4	59	21.91	21.35
KJ5	25	24.31	23.13
KJ6	32	24.14	23.14
KJ7	48	30.54	27.98
	44 ± 15	25.81 ± 3.25	24.60 ± 2.60

*Note*: BMI is body mass index.

## Data Availability

Not applicable.
